# Practitioners’ and Policymakers’ Successes, Challenges, Innovations, and Learning in Promoting Children’s Well-being During COVID-19: Protocol for a Multinational Smartphone App Survey

**DOI:** 10.2196/31013

**Published:** 2021-07-29

**Authors:** Jennifer C Davidson, Dimitar Karadzhov, Graham Wilson

**Affiliations:** 1 Institute for Inspiring Children's Futures School of Social Work and Social Policy University of Strathclyde Glasgow United Kingdom; 2 Digital Health & Wellness Group Department of Computer & Information Sciences University of Strathclyde Glasgow United Kingdom

**Keywords:** mobile phones, smartphone app, qualitative, mixed method, international, survey, service providers, policy, practice, children’s rights, well-being, COVID-19, pandemic, app, mHealth, children

## Abstract

**Background:**

The advent of COVID-19 abruptly thrust the health and safety of children and families into greater risk around the world. As regional and local governments, nongovernmental organizations, communities, families, and children grapple with the immediate public health impact of COVID-19, the rights and well-being of children, especially those who are already marginalized, have been overlooked. Those working with children have likely encountered unprecedented challenges and responded in innovative ways in efforts to address the needs and rights of all children.

**Objective:**

This paper presents a protocol for a large-scale, multinational study using a new smartphone app to capture the real-time experiences and perspectives of practitioners and policymakers supporting children and families during the COVID-19 pandemic around the globe in relation to a children’s human rights *4P* framework of protection, provision, prevention, and participation.

**Methods:**

This protocol describes a mixed methods survey utilizing a custom-built iOS and Android smartphone app called the COVID 4P Log for Children’s Wellbeing, which was developed in close consultation with 17 international key partner organizations. Practitioners and policymakers working with and for children’s well-being across 29 countries and 5 continents were invited to download the app and respond to questions over the course of 8 weeks. The anticipated large amount of qualitative and quantitative response data will be analyzed using content analysis, descriptive statistics, and word frequencies.

**Results:**

Formal data collection took place from October 2020 until March 2021. Data analysis was completed in July 2021.

**Conclusions:**

The findings will directly inform the understanding of the ways in which COVID-19 has impacted practitioners’, managers’, and policymakers’ efforts to support children’s well-being in their practices, services, and policies, respectively. Innovative and ambitious in its scope and use of smartphone technology, this project also aims to inform and inspire future multinational research using app-based methodologies—the demand for which is likely to continue to dramatically rise in the COVID-19 era. Mitigating the risks of longitudinal remote data collection will help maximize the acceptability of the app, respondents’ sustained engagement, and data quality.

**International Registered Report Identifier (IRRID):**

DERR1-10.2196/31013

## Introduction

### Background

The advent of COVID-19 abruptly thrust the health and safety of children and families into greater risk around the world [1-3], with far-reaching consequences for public health, child protection, peace, and justice, globally. While focusing on mitigating the immediate public health and economic impacts of the pandemic, regional and local governments, communities, and families may risk overlooking its acute and long-lasting effects on the rights and well-being of children, in particular, those who are already marginalized [4]. Agencies worldwide have recognized that minimizing the negative impact of the COVID-19 emergency on children—in particular, those related to public health responses—will be critical to protecting children’s well-being. This will also be essential to realizing United Nations (UN) Agenda 2030 and Sustainable Development Goals [5].

The distinctive impact of this pandemic on children is vast, and the risks posed to children’s rights to survival and development, as well as to their rights to special protection, education, health, and food, have been greatly compounded by COVID-19, and in many cases, by governmental priorities and responses [6-8]. Children’s rights to participate in decisions that impact them have also likely been compromised [9,10]. For those children who are in detention, in alternative care, in migration—especially those who are unaccompanied—and living in poverty, this pandemic and related measures of confinement have likely had an even greater impact. A distinctive and well-coordinated response is required by governments, nongovernmental organizations, and local communities to mitigate these impacts [2,11].

Supporting children by implementing policy and practice responses that are focused on distinctly promoting children’s well-being will form a part of this necessary response, throughout all stages of this pandemic. In some cases, given the changed nature of their work and the constraints faced by practitioners and policymakers alike, these approaches will need to be innovative and may be unprecedented. A better understanding of these circumstances across cultures, countries, and continents is essential to address the impact on children now and in the medium term [2,3].

### Utility of Smartphones for Capturing Critical Information from Hard-to-Reach Groups

The COVID-19 pandemic has posed unparalleled challenges to the conduct of traditional face-to-face research [12]. Harnessing the capabilities of mobile phone devices has become the cornerstone of innovative research methodologies for the remote collection of qualitative and quantitative data, including in low- and middle-income countries, during this time [12].

The use of mobile phones for gathering qualitative and quantitative data across a range of geographical settings has been demonstrated to be feasible and effective [13,14]. Mobile phones are flexible, affordable, and naturalistic devices, which makes them a powerful tool for generating rich, highly contextualized insights, including in hard-to-reach or vulnerable populations [15-18].

Beyond enabling the generation of substantive findings in the health and social sciences, certain mobile methods such as mobile phone diaries and other free-text response formats have shown intrinsic benefits for study participants [14,19]. Examples include increased autonomy, enhanced opportunities for self-expression and reflection, and more acceptable communication of sensitive topics and in high-stress environments [12,14].

### COVID 4P Log for Children’s Wellbeing Smartphone App for Conducting Global Research

In response to the need to capture vital, time-critical cross-country data in the midst of this global emergency, the research team designed a smartphone app–based survey to explore how children’s rights and well-being are being supported in this pandemic. To generate insights that would inform policies and practices during ongoing and future stages of COVID-19 and in preparation for future public health emergencies, we set out to understand effective practices and policies, challenges, innovations, lessons learned, and recommendations for improving practice and policy in relation to the protection, prevention, participation, promotion, and service provision for children’s rights and well-being around the globe.

Practitioners and policymakers working with and for children’s well-being across 29 countries and 5 continents were invited to download the app and respond to questions over 8 weeks. To do so effectively, we established a broad partnership of 17 international key partners, whose roles span intergovernmental policymaking, child rights advocacy, workforce capacity-building, service delivery, and monitoring roles at UN level. With their active involvement, the team designed a novel fast-capture smartphone app called the COVID 4P Log for Children’s Wellbeing [20].

As well as gathering practice- and policy-related data on key aspects of children’s lives and rights affected during this pandemic, the daily question schedule and the longitudinal nature of the survey were intended to provide a reflection space for respondents to share their achievements, challenges, and concerns. In addition to the core questions, a series of questions enquires about respondents’ own coping and well-being during the pandemic; this component of the survey was influenced by diary-based research, in that it attempted to capture data in the form of intimate reflections or confessionals on these topics, which might be sensitive or difficult to discuss [19]. Engaging in such written reflections may also have intrinsic personal benefits to the respondents, by having the opportunity to share and be heard [19]. In light of the public health containment measures and other mobility restrictions which have been forcing many professionals into remote lone working, as well as the undue increases in the safety risks and workloads for many frontline workers worldwide, offering professionals a space to share their concerns, successes, and reflections is likely to increase the acceptability and ethical sensitivity of the research [19,21,22].

### Aims

The aim of this paper is to provide researchers with insights into the design decisions and approaches undertaken within this project and to contribute to the growing evidence base on the use of fast-capture digital technologies for mixed methods research with hard-to-reach groups.

## Methods

### Research Design

This project is a mixed methods study utilizing a smartphone app–based survey to enable a fast-capture, contextually aware, and unobtrusive approach to remote data collection.

### Research Team

The diverse and complementary expertise of the research team and the relationships with partner organizations and other stakeholders have been critical to the project’s success. The research team comprises an international expert in children’s rights and well-being, with a wide range of international policy and practice stakeholder collaborations; an expert in human–computer interaction, with experience of user-led app development; a data manager, with experience working with Microsoft Azure databases and app-based research projects; an experienced contracted app developer; a researcher with experience of app-based data collection with hard-to-reach groups; and 2 knowledge exchange administrators and research assistants with communication, visual design, and stakeholder liaison experience.

### Sampling and Recruitment

Eligible participants were adults (aged 18 years of age or above) working in a role supporting children’s well-being, such as a policymaker, a practitioner, a supervisor, or a manager, in a paid or voluntary capacity, with sufficient English language fluency to engage with the app.

Remote participant recruitment and data collection pose distinct challenges to achieving diverse, representative samples [12]. The pragmatic combination of purposive (maximum variation), convenience and snowball sampling strategies in this study aimed to ensure the efficient collection of rapid, time-sensitive insights from a diverse cohort of professionals amid a global emergency [12,23,24]. Purposive sampling ensured that respondents from a wide range of countries, regions, roles, and areas of work were encouraged to participate. The snowball and convenience sampling approaches relied on the key partners, who assisted with recruitment and follow-up. Snowball sampling was relied upon because study participants, such as supervisors and service managers, were encouraged to disseminate information about the study across their teams, organizations, and sectors.

Each key partner assisted with recruitment by proposing the countries in which their organizational networks had the greatest reach and influence and in which they believed they might be able to recruit at least 50 respondents. In determining the target sample size, we anticipated that achieving this level of representation from all 29 target countries would be challenging and that there would be a reduction in the numbers of participants sustained throughout the 8-week logging period, given the level of participant retention in other surveys [12].

The research team and the key partners promoted the study via social media, the project website, e-newsletters, and videos. Interested participants had the option of visiting the project website or downloading the app directly into their smartphone (Google Play or Apple App Store). Monetary incentives for participation were not offered in order to minimize the risk of coercion and due to the purposive sampling strategy, which mainly relied upon existing networks and relationships.

In the first phase, the Android or iOS app was available for free download to users in the following countries: Australia, Belgium, Canada, Finland, Greece, India, Iraq, Israel, Italy, Jordan, Kenya, Lebanon, Malawi, the Netherlands, New Zealand, Norway, Palestine, the Philippines, Romania, Sierra Leone, South Africa, Sweden, the United Kingdom, and the United States. It was also made available in the Google Play Store’s *rest of the world* category in order to recruit app users in Eswatini, Ethiopia, Tanzania, Uganda, and Zambia. The app was only available in English. After consulting with the key partners, it was decided that it would not be made available in Latin America or conflict-affected countries. Furthermore, users with a smartphone having Android versions older than 8.0 (Oreo) or iOS versions that are older than 12.5, and those whose smartphones run on operating systems other than Android and iOS were not able to take part.

The app was available to download for 3 months in its first phase from October 7, 2020 to 5 January 5, 2021, in order to capture data during the pandemic’s second wave in many countries [25]. The app was removed from the Google Play and App Stores on January 5, 2021. Because some participants had just begun their 8 weeks of questions at that time, the data collection concluded 8 weeks later on March 9, 2021.

### Data Collection

#### Smartphone App Design

The app was built for both Google’s Android (version 8 and above) and Apple’s iOS (version 12.5) using React Native (Facebook Inc). The smartphone app was developed in partnership with an independent app developer contracted by the University of Strathclyde, which enabled a flexible tailored approach and delivered a quality product [26]. It is free to download, small in size, and takes little processing and battery power.

To ensure the acceptability and accessibility of the app, an app development steering group was formed, comprising representatives from the key partner organizations. The steering group advised on the app logo, other design features, and the survey questions, as well as taking part in the test flight phase.

Several changes were made as a result of the test flight feedback, including fixing technical issues and adjusting minor aspects of the wording, layout, and flow (sequence of task steps). To increase motivation as well as flow through the app, after submitting a response, a screen was added thanking the participant for completing a response that day, and a certificate of contribution was offered to those who completed questions across all 8 weeks. Consultations with key partners also led to the use of a more vibrant color scheme for the project’s visual identity.

There were 6 main components of the app: Onboarding, Daily Log, Calendar, FAQ (frequently asked questions), Information (about the project), and Settings. Calendar, FAQ, Information, and Settings were all accessible via a navigation bar at the bottom of the app.

#### Onboarding

**Figure 1:**
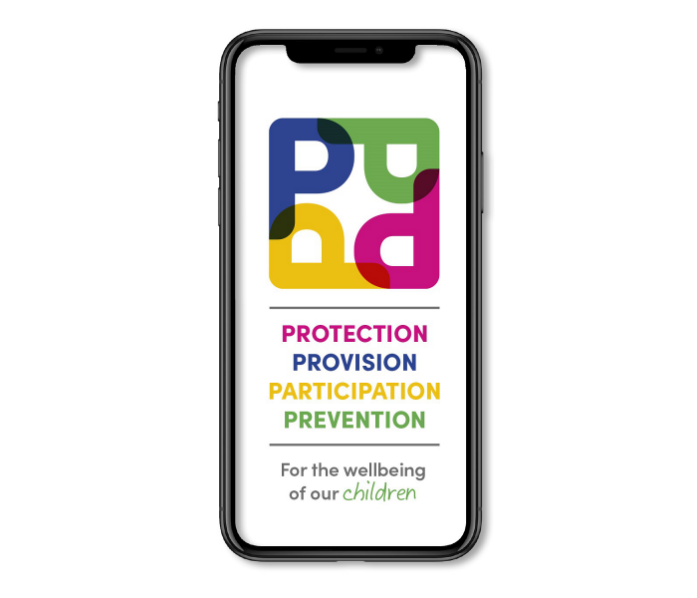
Loading screen with app logo.

**Figure 2:**
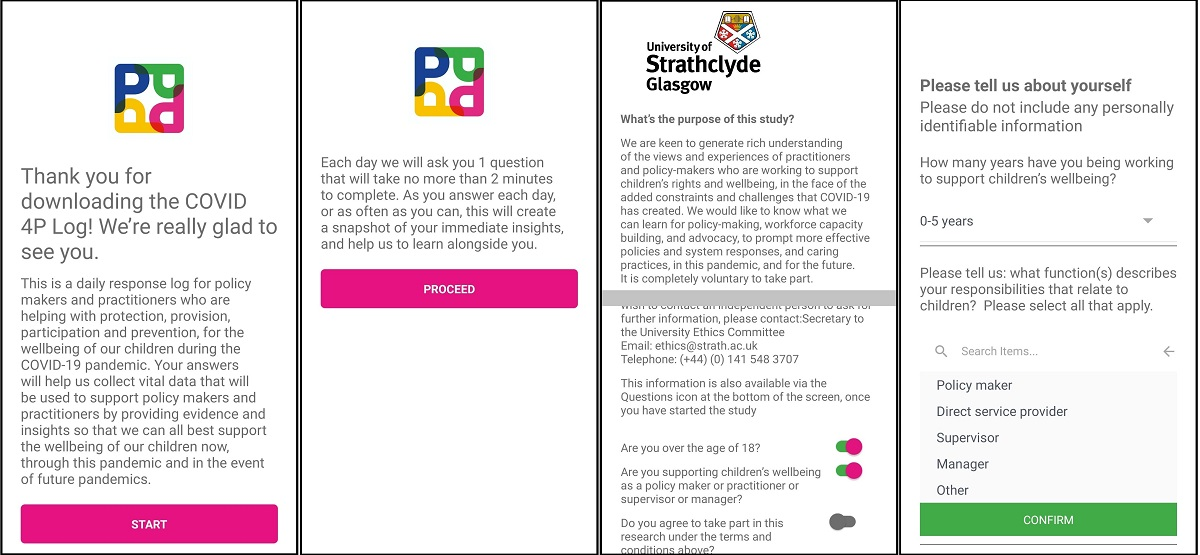
The app onboarding process (from left to right): initial welcome screen, message to user, consent form and agreement indicators, and demographic questionnaire.

Onboarding is typically the process of welcoming a new user and introducing them to how the app works. For our app, after a branded loading screen (Figure 1), the onboarding text (Figure 2) first thanked the person for downloading the app and briefly explained for what it would be used—a daily response log to record policymakers’ and practitioners’ insights and experiences. The next screen informed the user that they would only be asked 1 question per day, which would take no more than 2 minutes to answer. The user was then provided with an information and consent screen (terms and conditions), which fully explained the design and purpose of the research study, data governance, anonymity, and project partners. To indicate that they consented to taking part, the user was asked to confirm, by tapping on a button next to each statement, that they were over the age of 18, were working in an appropriate role related to children’s well-being, and agreed to the above terms and conditions. The subsequent screen was used to record information about the participant’s professional role and experience. At the end of onboarding, the participants were taken to their first Daily Log screen.

#### Daily Log

Each day, the app displayed a single Daily Log screen when the participant first opened the app. It showed the question that had been assigned to that particular day and provided a free-text box for the participant to write as much or as little as they wanted. The screen also had an optional question asking the participant if they wanted to provide any general or separate insights or comments. The answers were recorded when the *Submit* button was clicked.

#### Calendar

**Figure 3:**
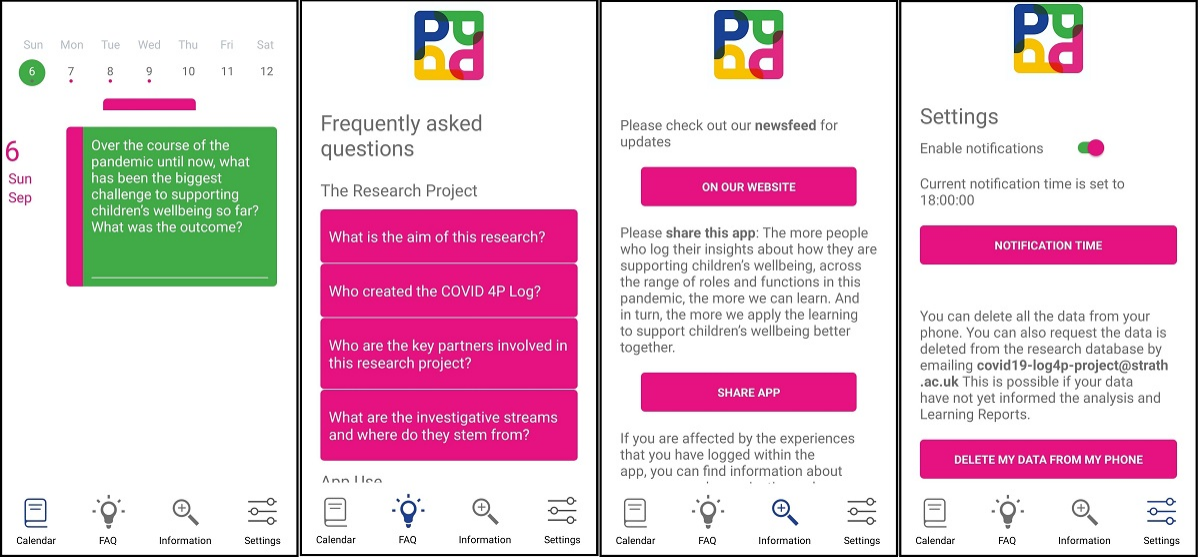
Main app screens (from left to right): Calendar, FAQ (frequently asked questions), Information, and Settings.

Participants were given the ability to revisit answers that they had provided over the previous weeks, in case they wanted to amend what they had said. They were also able to answer the given week’s 7 questions at any time, if they did not want to answer daily. The calendar (Figure 3) showed the days of the week at the top of the screen, and each day with a question had a pink dot. By selecting the day, the question and any previous answer were displayed in the lower part of the screen, and these text boxes could be edited to change the answer. Tapping on the pink bar under the days of the week displayed a larger month-long calendar.

#### FAQ

The FAQ screen (Figure 3) provided information about the research project, how to use the app, data protection, investigator contact details, and terms and conditions.

#### Project Information

This screen provided a link to the main project website, a button to share a link to the app and project information videos via social media platforms, and links to country-specific resources and organizations, should they wish to seek help for a child or if they were emotionally affected as part of their involvement in the project.

#### Settings

The Settings screen allowed participants to enable or disable notifications and set the time at which the Daily Log reminder notification was sent to them. For privacy and data governance reasons, it also contained a button that would delete all the data they had provided stored locally on the device. If they wished to have their data removed from the main research database, they could request this via a project email address.

#### Survey Development

The survey was informed by 8 investigative streams, and a new set of questions corresponding to each investigative stream was introduced each week (Table 1). These streams were shaped by a 4P (protection, provision, prevention, and participation) children’s human rights framework designed to better understand the ways practitioners and policymakers were able to protect children, provide for their unique needs, enable their participation in decisions that affect them, and prevent harm in this pandemic context. While limited in specificity, the 4Ps nevertheless offer accessible framing of the rights most closely corresponding to aspects of children’s well-being that are the focus of this study. The benefits and drawbacks of both human rights and well-being approaches were considered by the key partners [27]. Both rights and well-being were chosen in order to most effectively address the wide range of interests that shape the key partners’ varied work.

The survey questions were drawn from a wide range of mainly COVID-related multidisciplinary international policy- and practice-focused English-language publications, particularly those issued by established international institutions concerned with children, including UNICEF [28], the UN Committee on the Rights of the Child [29], and the Pathfinders for Peaceful, Just, and Inclusive Societies [30]. These publications are mainly grey international policy and practice literature, where concerns about children during COVID-19 were shared earlier in the pandemic than they were in the peer-reviewed academic literature.

In focus groups, key partners contributed their feedback on selected questions across the investigative streams, reflecting on clarity, situational and cultural relevance, sensitivity, and overall acceptability of the questions. In addition, individual key partners identified specific aspects of children’s lives for inquiry relevant to their organizational focus. In some cases, specific questions were drafted, shared, and further adapted in partnership. Additionally, an international group of child researchers involved in advising the Life under Coronavirus international peer research project [31] met as a focus group to directly advise the research team on a specific set of questions to inform to the investigative stream on children’s participation.

**Table 1:** Survey schedule.

Topic (investigative stream)			Example survey question		Survey items, n
**Week 1**					
	Onboarding (demographic and work-related) questions	What best describes what you do in relation to children?		11	
	Learning from the pandemic so far	What has gone well in your sector’s support of children’s well-being during COVID-19?		14	
**Week 2**					
	Protection: Ending violence against children	Has your sector experienced challenges in protecting children from violence during COVID-19?		21	
	Respondents’ coping and well-being	On a scale of 1-5, how stressed and anxious have you been feeling in the past week?		5	
	App evaluation	How would you describe your overall experience of taking part in this study so far?		5	
**Week 3**					
	Provision: Access to food, health, education	How have you changed what you do in your work because children have had more restricted access to basic needs, eg, food, education and health care, during COVID-19?		21	
**Week 4**					
	Collaborations, flexibility, transparency, and trust: Applying evidence from past emergencies to COVID-19	In past emergencies around the world, government actions that facilitate trust, connections and collaborative working between government, across sectors and within communities, have been found to be important for recovery from the emergency. Have you seen these actions by your government(s) in this pandemic?		17	
	App evaluation	Has taking part in this study had an impact on your work?		4	
**Week 5**					
	Prevention: Children’s social and emotional well-being	Have you found that children have experienced mental health issues during COVID-19?		17	
**Week 6**					
	Special considerations - Access to justice, alternative care, disabilities	Children in detention are likely to be in poorer health than those who are not. The COVID-19 outbreak exacerbates the challenges these children face. Have children been released from detention so that they can return to their families and self-isolate?		15	
	App evaluation	What has been motivating you to continue taking part in this project?		4	
**Week 7**					
	Children’s participation	During COVID-19, have children’s views been sought about policy or practice decisions that affect their lives?		21	
**Week 8**					
	Preparing to rebuild post–COVID-19	What are the priorities for children that should be emphasized following the first phases of COVID-19? Please tell us more.		15	
	App evaluation	If you could, would you want to keep using an app of this sort as an ongoing part of your day-to-day work?		7	

#### Survey Structure

App respondents were asked a total of 177 questions over a period of 8 weeks (Table 1). On average, 1 main open-ended question with 3 follow-up questions (open or closed-ended) were asked each day. Week 1 began with a series of onboarding questions about respondents’ demographic and work-related information such as gender, country and region of work, years of experience, professional role, and sector. In addition to questions related to the investigative streams, a series of questions was also asked about respondents’ own coping and well-being, as well as about their experience with the app.

As soon as questions were completed, they were automatically uploaded to the cloud server when Wi-Fi or mobile data were available. At the conclusion of participants’ 8 weeks of responses, the app provided them with a project email address and offered the opportunity to give theirs, if they wished to stay in touch with the project and receive updates.

### Data Management

A data management plan was completed in line with university standards, and the data manager ensured compliance with the plan. The project data (survey responses) were transmitted to a dedicated, European Union–based cloud-hosted database at OVHCloud (OVH Groupe SAS); this occurred when the research participants submitted their responses as soon as Wi-Fi or mobile data access was available. The data were then accessed with DBeaver (open source) database management software and extracted as a .csv file. Data were stored on the university’s internal systems and were clearly versioned. The data were secured by having a passkey and by only being accessible from specified IP addresses.

After data were extracted from the database in .csv format, they were stored in the university’s internal networked storage in a location only accessible to the research team. The data manager produced a script in Excel using Visual Basic script (Microsoft Inc) to transform the data into a usable format. This was necessary to overcome the challenges of working with large amounts of data.

All participant data were strictly anonymous. There were no personally identifiable data collected within the app by default, and users were given instructions not to provide any personally identifiable information within the open-text responses. Prior to data analysis, the data manager screened the data to ensure that no personally identifiable information had inadvertently been provided. All such data were anonymized; access to the raw data was restricted to the research team. All data are stored on Azure cloud storage (Microsoft Inc) in full compliance with the General Data Protection Regulation legislation.

### Data Analysis Plan

The open-ended survey response data will be analyzed using qualitative content analysis, which involves open coding, grouping, categorization, abstraction, and conceptual mapping [32]. The coding strategy will involve both structural and data-driven (inductive) approaches [33,34]. The structural coding will be based upon the investigative streams underpinning this study, as well as upon the survey questions. The responses to the closed-ended survey questions will be analyzed using descriptive statistics. Cross-tabulation will be performed to compare participants’ responses according to sociodemographic characteristics such as country, professional role, gender, experience level, and others.

The qualitative data analysis software, NVivo (version 12; QSR International), will be used to assist with the data analysis. NVivo enables the efficient and systematic storage, management, analysis, and sharing of large amounts of qualitative and quantitative data [35-37]. Various data visualization, coding, and text mining features—such as word clouds, word frequency queries, text search queries, word trees, coding context, and matrices—will be used to facilitate the efficient generation of rich insights from the data [36].

Throughout this process, the researchers will keep a research diary containing both methodological and analytic memos [32]. Regular team meetings will be conducted to conduct formal and informal coding comparisons, discuss emerging codes and themes, clarify ambiguous or unclear datapoints (such as professional jargon, abbreviations and vernacular), and help minimize personal and professional biases that could inadvertently affect the analysis [38].

### Research Advisory Group

After data collection, the key partners will be invited to a research advisory group, which is intended to comprise a representative sample of stakeholders including policymakers, child rights advocates, and service providers who will be asked to comment on an accessible summary of the anonymized findings. Their input will help articulate the implications of the findings for policy and practice. Additionally, Life under Coronavirus child researchers will be invited to reflect with the research team on the study’s conclusions. This approach to member checking will enhance the credibility and trustworthiness of the findings [39].

### Ethical Considerations

Conducting research that targets at-risk or hard-to-reach groups, including those working in high-stress environments, during a global pandemic raises acute ethical concerns [12]. In this study, the potential risks of participation—such as the undue time and emotional commitment required and the loss of privacy—were evaluated in light of the considerable expected benefits of promoting child welfare and informing policy and practice worldwide. Several procedures, outlined below, were undertaken to minimize the risks for and burden on study participants and to promote their engagement.

Information on how data are gathered and used during the project was provided to participants on Participant Information and Consent Form pages, to which respondents were required to agree prior to accessing the app questions. This was also available within the FAQ pages in the app itself and on the project website (a link was provided in the Information section). Participants were able to withdraw from the study by stopping participation and removing the app from their mobile phone or by contacting the study administrator by email (which was explained in the Information section of the app). They could remove any data stored on the phone via a button in the Settings or by deleting the app from their phone.

There was concern about the risk of financial costs incurred as a result of using a smartphone app, especially given that participants from low-income countries were involved in this study. The app would have been fully free for the participant to use if they used a Wi-Fi connection for which they were not financially liable (eg, public or workplace) or that had an unlimited data allowance. Users who relied upon mobile data connections or personal Wi-Fi with a data usage cap, however, may have incurred a personal cost for taking part. All efforts were made to minimize the size of the app download; it requires 37.6 MB (Android) or 21.1 MB (iOS) of data for initial download. When bugs needed to be fixed, however, some participants would have been required to download updates of a similar size.

The risk of placing undue demands on participants was addressed in the design of the app and the survey. The app promoted autonomy by allowing participants to initiate the activity themselves, to determine when and if they wished to be notified by the app to complete the daily question, and to decide if they preferred to complete more than 1 daily question at a time by using the Calendar setting. Participants could also choose to not answer questions. There was a voice-to-text option for those who preferred to speak (and amend) rather than type their responses. Participants could begin and end their participation at a time that suited them.

It is important that the study upheld a duty of care to participants, within the bounds of an anonymous study, as the questions explored how policies, services, and professional practice might or might not be meeting children’s needs. It was anticipated that some participants might experience or would express concerns about, increased risks of harm to children; therefore, the app directed participants to information on the project website about where in their country they could seek support for their concerns about a child or for their own mental health.

## Results

The study was initiated in April 2020, and the research team began liaising with key partners in May 2020. App development and initial testing were undertaken between June and August 2020. The test flight process began in August 2020, and the app and the majority of questions were finalized in September 2020. Data collection was undertaken between October 2020 and March 2021. Data analysis was completed in July 2021. Dissemination to policy and practice audiences, as a first priority, and to others, later on, will be planned with key partners, given the time-sensitive nature of the findings and the urgency of the issue of children’s well-being. Learning reports will be published from June 2021 onward. Key partners will play a further and pivotal role throughout the dissemination stages.

## Discussion

### General

This paper presents the protocol for a smartphone app–based survey with a distinctive global scope, a participatory approach to survey development involving a diverse group of partner organizations, and a time-sensitive focus on practitioners and policymakers working across a range of settings, countries, continents, and cultures during the COVID-19 pandemic. Ultimately, this study aims to explore a range of concerns at practice, service, and policy levels that reflect the complexity of children’s lives and the profound implications of this global emergency.

The role and commitment of the key partners to this project have made a central contribution to its reach, inclusivity, and rigor. Their involvement has been underpinned by goodwill, positive relationships with the research team, and a collective sense of urgency about the issues facing children at this time.

### Study Limitations and Risks

There are a number of risks and limitations in this study [12,18].

#### Linguistic and Cultural Accessibility

The app was only available in English. This likely posed challenges to the engagement of persons with low literacy levels or lack of fluency in English. Crucially, study participation was contingent upon smartphone ownership and the availability of Wi-Fi smartphone devices; it was not feasible to provide data packages.

#### Data Integrity and Quality Risks

We anticipate typing errors and single-word responses, ambiguous vernacular, unwillingness to enter long responses, and difficulties using the voice-to-text feature. Some responses will thus lack sufficient context or elaboration. Additionally, due to the inherent limitations of anonymous survey designs, we were unable to ask participants to elaborate or clarify responses. Conversely, participants were not able to clarify any questions via the app.

Ensuring data integrity is another challenge of using an anonymous survey format. Because we were not able to ascertain how many users were actively using the app, it was difficult to ensure that all data were being accurately received. To address this, during the beta testing phase and periodically during the early live run of the app, specifically identified researchers and partners filled in the responses using only their initials, allowing the data manager to check to ensure data were being received as expected.

#### Participant Attrition, Engagement, and Motivation

Some general challenges related to the use of a longitudinal smartphone app survey warrant discussion. Participants may have forgotten to answer the daily questions or lost motivation over the 8-week period. The high burden of participation (177 questions asked over 8 weeks) is likely to have increased attrition. In addition, the relevance of the questions to participants may have varied between investigative streams, given the range of participants’ roles, experience, and knowledge, which may also have contributed to attrition. And, due to the nature of their work, participants may have lacked the time or the privacy to sustain detailed daily responses. The resulting attrition may have led to survey questions in the latter weeks of the study being insufficiently addressed. Furthermore, despite anonymous data collection, some prospective participants may have concerns regarding data privacy and anonymity. The remote and anonymous recruitment and data collection will make it more challenging to establish rapport; this may have contributed further to attrition and to reduced respondent motivation.

#### Technological Risks

The creation of an entirely new app, over an established app service, introduces potential risks such as technical malfunctions and compatibility issues across devices. Technical malfunctions can hinder a user from being able to use the features of the app as intended, which could lead to (among other issues) loss of data, if there are data entry or upload malfunctions; inaccessible information or confusion about how to use the app, if the FAQ or Information sections malfunction; and reduced trust and increased frustration with the app and project, leading to reduced use of the app or even removal of the app from the user’s phone.

#### Risks to the Transferability of the Study Findings

Snowball and convenience sampling strategies in this project have likely resulted in nonrepresentative samples [12]. The sample was purposefully restricted to a set of target countries that did not include humanitarian or conflict settings or countries in fragile states. Furthermore, there may be vast differences among the response rates by individual participants. This may also negatively impact data transferability.

### Conclusion

The findings of this global smartphone app–based survey study will directly inform understanding of the ways COVID-19 has impacted practitioners’, managers’, and policymakers’ efforts to support children’s well-being in their practices, services, and policies, respectively. This knowledge will be critical to leveraging learning and innovations to better protect children, provide for their unique needs, prevent negative long-term impacts of the pandemic on their well-being, and enable their participation in decisions that affect them. The project also aims to inform the development of a range of publications, best practice guidelines, and other outputs focused on improving pandemic-related professional practices, child rights–oriented policies, and future applications of a smartphone app methodology for real-time responses. Mitigating the risks of longitudinal remote data collection will help maximize the acceptability of the app, respondents’ sustained engagement, and data quality.

## Abbreviations

4P: protection, provision, prevention, and participation
FAQ: frequently asked questions
UN: United Nations
